# Effects of dapagliflozin on myocardial motion parameters and cardiac electrophysiology in patients with type 2 diabetes Mellitus and coronary heart disease

**DOI:** 10.3389/fcvm.2026.1745362

**Published:** 2026-06-04

**Authors:** Fang Liu, Rui Jin

**Affiliations:** 1 Department of Cardiology, Dalian University Affiliated Xinhua Hospital, Dalian, Liaoning Province, China; 2 Department of Respiratory Medicine, Dalian University Affiliated Xinhua Hospital, Dalian, Liaoning Province, China

**Keywords:** cardiac function, coronary heart disease, dapagliflozin, exercise tolerance, myocardial strain, type 2 diabetes mellitus

## Abstract

**Background:**

Type 2 diabetes mellitus (T2DM) and coronary heart disease (CHD) often coexist and exacerbate cardiac dysfunction. This study aimed to evaluate the effects of dapagliflozin on myocardial motion parameters and cardiac electrophysiology in patients with T2DM and CHD.

**Methods:**

This retrospective study included 262 patients with T2DM and CHD admitted between January 2021 and January 2025. Patients were assigned to either a conventional treatment group (*n* = 137; standard CHD therapy plus metformin) or a dapagliflozin group (*n* = 125; standard CHD therapy, metformin, and dapagliflozin 10 mg daily), with both groups treated for 4 weeks. Assessments included echocardiographic cardiac function, myocardial motion parameters, left ventricular pressure-strain loop (LV-PSL) indices, electrophysiological activity, exercise tolerance, and adverse cardiovascular events.

**Results:**

Baseline characteristics were comparable between groups. The dapagliflozin group demonstrated superior clinical efficacy (93.60% vs. 76.64%, *P* < 0.001) and greater improvements in left ventricular ejection fraction, myocardial strain and work indices, and exercise tolerance (all *P* < 0.01). Dapagliflozin was also associated with enhanced cardiac electrophysiological activity, including longer QT and Tp-Te intervals and reduced ST depression (all *P* < 0.01). Adverse event rates did not differ significantly between groups.

**Conclusion:**

In patients with T2DM and CHD, adding dapagliflozin to conventional therapy improved cardiac function, myocardial performance, electrophysiological parameters, and exercise tolerance without increasing adverse cardiovascular events.

## 1 Introduction

Type 2 diabetes mellitus (T2DM) continues to represent a substantial and escalating global health challenge, with prevalence rates rising in tandem with increases in obesity, sedentary lifestyles, and population aging (1). Patients with T2DM are at a markedly heightened risk for cardiovascular complications, most notably coronary heart disease (CHD), which remains the principal cause of morbidity and mortality in this population (2). The pathophysiological interplay between T2DM and CHD is multifaceted, involving chronic hyperglycemia, insulin resistance, dyslipidemia, endothelial dysfunction, subclinical inflammation, oxidative stress, and accelerated atherogenesis (3). These intertwined mechanisms not only accelerate the progression of macrovascular disease but also promote adverse cardiac remodeling and impair myocardial function, culminating in heart failure and other cardiac complications (4).

Conventional management strategies for patients with T2DM and coexisting CHD have primarily focused on stringent glycemic control and risk factor modification, including optimization of blood pressure, lipid profiles, and lifestyle interventions (5). While metformin remains the foundational antihyperglycemic agent due to its extensive evidence of cardiovascular safety and efficacy, a significant residual risk for major adverse cardiovascular events persists even among patients achieving adequate glycemic targets (6). This residual risk highlights the need for adjunctive therapies that can exert beneficial effects on cardiovascular outcomes through pleiotropic mechanisms in addition to glucose lowering (7).

In this context, sodium-glucose cotransporter 2 inhibitors (SGLT2i), a novel class of antihyperglycemic agents, have emerged as a promising therapeutic approach (8). SGLT2i such as dapagliflozin promote urinary glucose excretion by targeting SGLT2 in the proximal renal tubules, resulting in reductions in plasma glucose independent of insulin secretion (9). Beyond their glucose-lowering capability, large-scale cardiovascular outcomes trials have demonstrated that SGLT2i reduce the risk of hospitalization for heart failure and major cardiovascular events among patients with T2DM, including those with established atherosclerotic cardiovascular disease (10). These cardiovascular benefits have been attributed to diverse pathways involving hemodynamic modulation, reduction in arterial stiffness and preload/afterload, favorable effects on myocardial energy utilization, natriuresis, and attenuation of pathologic cardiac remodeling (11, 12).

Despite these advances, the precise impact of SGLT2i therapy on specific cardiac functional and electrophysiological parameters—particularly in T2DM patients with established CHD—remains incompletely elucidated (12). Myocardial motion parameters, as assessed using echocardiography-based speckle tracking and pressure-strain loop (PSL) analyses, provide sensitive and reproducible markers of subclinical myocardial dysfunction and are predictive of future adverse cardiovascular outcomes (7). Similarly, disturbances in cardiac electrophysiology, including alterations in QT interval, Tp-Te interval, and ST-segment depression, are well-recognized harbingers of ventricular arrhythmia and sudden cardiac death in this population (13). However, data detailing the modulatory effects of SGLT2i agents on myocardial motion and electrophysiological indices in the context of T2DM complicated by CHD remain scarce (13).

Recent evidence suggests that SGLT2i may directly influence myocardial cellular metabolism, promote reversal of adverse myocardial remodeling, and enhance cardiac efficiency by altering substrate utilization—shifting myocardial metabolism toward more energetically favorable ketone bodies and free fatty acids, thereby improving adenosine triphosphate (ATP) generation per oxygen molecule consumed (9). Experimental studies further implicate these agents in the inhibition of sodium-hydrogen exchanger (NHE1) activity, reduction of intracellular sodium and calcium overload, preservation of mitochondrial integrity, and attenuation of fibrosis and inflammation—all of which can affect both contractile function and electrical stability of the myocardium (9, 12).

Given these mechanistic insights and the potential for SGLT2i to reshape the cardiovascular risk landscape in diabetes care, rigorous clinical data characterizing their effects on advanced cardiac function and electrophysiology are urgently needed (14). Such evidence is particularly relevant in the high-risk cohort of patients with coexistent T2DM and CHD, where optimal cardiac protection remains elusive despite contemporary guideline-directed therapy (14).

Accordingly, the present study was designed to systematically evaluate the effects of dapagliflozin, in combination with metformin, on myocardial motion parameters and cardiac electrophysiological activity in patients with T2DM complicated by CHD. The primary endpoints of this study were the changes in myocardial motion parameters, including longitudinal pre-stretch peak strain (LPSS) and circumferential pre-stretch peak strain (CPSS), as well as cardiac electrophysiological parameters such as QT interval, Tp-Te interval, and ST-segment depression. Secondary endpoints included improvements in echocardiographic cardiac function, left ventricular pressure-strain loop indices, exercise tolerance, and the incidence of adverse cardiovascular events. Using comprehensive echocardiographic techniques and electrocardiographic assessments, we aimed to delineate the extent to which dapagliflozin confers incremental benefits beyond standard conventional therapy.

## 2 Materials and methods

### 2.1 Participants and grouping

A total of 262 patients with T2DM complicated with CHD admitted to our hospital from January 2021 to January 2025 were retrospectively studied. According to different treatment methods, they were divided into conventional treatment group (*n* = 137) and dapagliflozin group (*n* = 125).

Inclusion criteria: ① The patients were confirmed to meet the diagnostic criteria of T2DM complicated with CHD after clinical examination (15); ② age ≥18 years old; ④ Complete clinical data of patients.

Exclusion criteria: ① patients with unstable angina, acute myocardial infarction or previous heart bypass surgery; ② patients with severe liver, kidney and other organ dysfunction; ③ patients with chronic infection; ④ previous use of sodium-glucose cotransporter 2 inhibitors (SGLT2i); ⑤ patients with hematological diseases.

### 2.2 Treatment methods

Both groups of patients received standard CHD treatment protocols after admission (16). This included the use of aspirin enteric-coated tablets (Shenyang Aojina Pharmaceutical Co., Ltd., Approval No. H20065051) at 100 mg per dose, once daily; clopidogrel bisulfate tablets (Hangzhou Sanofi Pharmaceutical Co., Ltd., Approval No. H20171237) at 75 mg per dose, once daily; and ticagrelor tablets (Shanghai Hui Lun Jiangsu Pharmaceutical Co., Ltd., Approval No. H20193252) at 90 mg per dose, twice daily. Additionally, statin therapy was administered as part of the standard coronary heart disease protocol, with atorvastatin calcium tablets (Pfizer Pharmaceuticals Ltd., Approval No. H20051407) at a dose of 20 mg per dose, once daily, or rosuvastatin calcium tablets (AstraZeneca Pharmaceutical Co., Ltd., Approval No. H20160610) at a dose of 10 mg per dose, once daily, based on individual patient lipid profiles and clinical judgment.

On this basis, the conventional treatment group were administered metformin hydrochloride tablets (Beijing Jingfeng Pharmaceutical Group Co., Ltd., Approval No. H11021518, 0.25 g per tablet) orally at a dose of 0.5 g per dose, twice daily.

The dapagliflozin group patients received metformin treatment combined with dapagliflozin. The dosage and frequency of metformin hydrochloride tablets for the dapagliflozin group were the same as those for the conventional treatment group. Additionally, they were treated with dapagliflozin tablets [Shandong Lukang Pharmaceutical Co., Ltd., Approval No. H20213815, specification: 5 mg per tablet (based on C_21_H_25_ClO_6_)], taken orally at a dose of 10 mg per dose, once daily. Both groups were treated for a period of 4 weeks.

### 2.3 Data collection and outcome measures

(1) Baseline Data Collection: For all enrolled patients, baseline demographic information was collected from the medical record system.
(2) Cardiac Function: Before treatment and 4 weeks after treatment, an ultrasound system (EPIQ 7C, Philips, Netherlands) was used to measure left ventricular ejection fraction (LVEF), left ventricular end-diastolic diameter (LVEDD), left ventricular end-systolic diameter (LVESD), left anterior descending artery diameter (LAD), and interventricular septal thickness at diastole (IVSd).
(3) Myocardial Motion: A two-dimensional echocardiography system (EPIQ 7C, Philips, Netherlands) was employed to automatically track myocardial motion trajectories using speckle tracking echocardiography (STE) technology. Longitudinal pre-stretch peak strain (LPSS) and circumferential pre-stretch peak strain (CPSS) were measured before and after treatment.
(4) Electrophysiological Parameters: QT interval, Tp-Te interval, and ST-segment depression were assessed using a standard 12-lead electrocardiogram (Cardiovit AT-102 Plus, Schiller, Switzerland) before treatment and 4 weeks after treatment.
(5) Pressure-Strain Loop Indices: Global Longitudinal Strain (GLS) and other Pressure-Strain Loop indices including Global Wasted Work (GWW), Global Work Efficiency (GWE), Global Work Index (GWI), and Global Constructive Work (GCW) were measured by combining echocardiography (EPIQ 7C, Philips, Netherlands) with pressure-strain loop analysis software (QLAB Advanced Quantification Software, Philips Ultrasound, Netherlands) before and after treatment.
(6) Exercise Tolerance: Exercise tolerance tests (ETT) were conducted using a treadmill test system (Quinton Q-Stress, Mortara, USA) before and 4 weeks after treatment, simultaneously monitoring heart rate and metabolic equivalents (METs). Electrocardiograms (Cardiofax V, Nihon Kohden, Japan) were used to monitor cardiac responses, determining improvements in exercise capacity.
(7) Adverse Cardiovascular Events: Adverse cardiovascular events, including recurrent angina pectoris, myocardial infarction, heart failure, or cardiac death, were evaluated through clinical records combined with electrocardiograms (Cardiofax V, Nihon Kohden, Japan) and echocardiograms (EPIQ 7C, Philips, Netherlands).

### 2.4 Statistical analysis

The indicator data collected and organized in this study were processed and analyzed using SPSS 29.0 (IBM Corp., USA) statistical software. Measurement data were normally distributed and are presented as means ± standard deviations (x̅ ± s), with comparisons between groups performed using t-tests. Count data are presented as frequencies (percentages) and were compared using chi-square (*χ*^2^) tests. All tests were two-tailed, with *P* < 0.05 indicating statistically significant differences between groups.

## 3 Results

### 3.1 Demographic and basic data

The demographic and baseline characteristics of the study population are presented in Table 1. There were no statistically significant differences between the conventional treatment group and the dapagliflozin group regarding sex distribution, age, body mass index, history of smoking, alcohol consumption, hypertension, hyperlipidemia, resting heart rate, systolic and diastolic blood pressure, duration of T2DM, family history of diabetes, duration and family history of CHD, or New York Heart Association (NYHA) cardiac function classification. These results indicate that the two groups were comparable at baseline.

**Table 1:** Comparison of demographic and basic data between the two groups.

Variables	Conventional Treatment group (*n* = 137)	Dapagliflozin group (*n* = 125)	t/*χ*^2^	P
Gender			0.157	0.692
Male	80 (58.39%)	76 (60.80%)		
Female	57 (41.61%)	49 (39.20%)		
Age(years)	58.72 ± 3.87	58.29 ± 4.18	0.028	0.977
BMI	23.12 ± 8.27	23.08 ± 7.93	0.042	0.966
History of smoking	49 (35.77%)	37 (29.60%)	1.127	0.288
History of alcohol consumption	60 (43.80%)	58 (46.40%)	0.179	0.672
Hypertension	112 (81.75%)	105 (84.00%)	0.232	0.630
High blood lipids	107 (78.10%)	99 (79.20%)	0.047	0.829
Heart rate (bpm)	77.62 ± 12.25	76.83 ± 12.65	0.515	0.607
SBP (mmHg)	139.57 ± 12.18	140.45 ± 13.07	0.565	0.572
DBP (mmHg)	82.36 ± 12.10	79.94 ± 11.96	1.624	0.106
Duration of T2DM (years)	7.38 ± 2.89	7.52 ± 2.73	0.383	0.702
Family history of T2DM	56 (40.88%)	43 (34.40%)	1.166	0.280
CHD Duration (years)	3.26 ± 1.04	3.31 ± 1.28	0.401	0.689
Family history of CHD	52 (37.96%)	45 (36.00%)	0.107	0.743
NYHA Classification			0.042	0.838
Grade II	62 (45.26%)	55 (44.00%)		
Grade III	75 (54.74%)	70 (56.00%)		

BMI, body mass index; SBP, systolic blood pressure; DBP, diastolic blood pressure; T2DM, type 2 diabetes mellitus; CHD, coronary heart disease; NYHA, New York heart association.

### 3.2 Clinical efficacy

As shown in Table 2, the dapagliflozin group demonstrated significantly greater clinical efficacy compared with the conventional treatment group. The proportion of patients with a conspicuous effect was higher in the dapagliflozin group than in the conventional group (57.60% vs. 36.50%; *χ*2 = 11.699, *P* < 0.001), while the void of effect rate was markedly lower (6.40% vs. 23.35%; *χ*2 = 14.53, *P* < 0.001). The total effective rate in the dapagliflozin group was significantly higher than that in the conventional treatment group (93.60% vs. 76.64%; *χ*2 = 14.53, *P* < 0.001). The proportion of patients who got better was not significantly different between the two groups (*P* = 0.490). These results indicate that dapagliflozin provides a superior clinical benefit in this patient population.

**Table 2:** Comparison of clinical efficacy between the two groups.

Efficacy	Conventional Treatment group (*n* = 137)	Dapagliflozin group (*n* = 125)	χ^2^	P
Void of effect	32 (23.35%)	8 (6.40%)	14.53	< 0.001
Get better	55 (40.15%)	45 (36.00%)	0.476	0.490
Conspicuous effect	50 (36.50%)	72 (57.60%)	11.699	< 0.001
Total effective rate	105 (76.64%)	117 (93.60%)	14.53	< 0.001

### 3.3 Echocardiographic cardiac function parameters

As shown in Table 3, there were no statistically significant differences between the two groups in baseline cardiac function parameters, including LVEF, left ventricular end-diastolic diameter (LVEDD), left ventricular end-systolic diameter (LVESD), left atrial diameter (LAD), and interventricular septal diastolic diameter (IVSd) (all *P* > 0.05). After treatment, the dapagliflozin group demonstrated significantly greater improvement in LVEF compared with the conventional treatment group (63.54 ± 10.77% vs. 59.48 ± 10.63%; t = 3.072, *P* = 0.002). The dapagliflozin group also exhibited lower LVEDD (55.03 ± 10.22 mm vs. 59.18 ± 10.31 mm; t = 3.269, *P* = 0.001), lower LVESD (9.32 ± 2.21 mm vs. 10.15 ± 2.36 mm; t = 2.942, *P* = 0.004), reduced LAD (27.18 ± 3.77 mm vs. 30.05 ± 3.89 mm; t = 3.932, *P* < 0.001), and lower IVSd (9.33 ± 1.11 mm vs. 10.06 ± 1.17 mm; t = 5.151, *P* < 0.001) after treatment compared with the conventional group. These data indicate that dapagliflozin is associated with superior improvements in echocardiographic cardiac function among patients with T2DM and CHD.

**Table 3:** Comparison of echocardiographic cardiac function parameters between the two groups .

Parameters	Conventional Treatment group (*n* = 137)	Dapagliflozin group (*n* = 125)	t	P
LVEF (%)				
Before treatment	45.27 ± 10.52	45.19 ± 10.46	0.063	0.950
After treatment	59.48 ± 10.63	63.54 ± 10.77	3.072	0.002
LVEDD (mm)				
Before treatment	70.34 ± 10.26	70.42 ± 10.95	0.058	0.953
After treatment	59.18 ± 10.31	55.03 ± 10.22	3.269	0.001
LVESD (mm)				
Before treatment	15.48 ± 3.49	15.27 ± 3.24	0.511	0.610
After treatment	10.15 ± 2.36	9.32 ± 2.21	2.942	0.004
LAD (mm)				
Before treatment	32.48 ± 4.19	33.05 ± 4.08	1.113	0.267
After treatment	30.05 ± 3.89	27.18 ± 3.77	3.932	< 0.001
IVSd (mm)				
Before treatment	10.25 ± 1.32	10.54 ± 1.62	1.600	0.111
After treatment	10.06 ± 1.17	9.33 ± 1.11	5.151	< 0.001

LVEF, left ventricular ejection fraction; LVEDD, left ventricular end-diastolic diameter; LVESD, left ventricular end-systolic diameter; LAD, left atrial diameter; IVSd, interventricular septal diastolic diameter.

### 3.4 Left ventricular myocardial motion parameters

**Figure 1:**
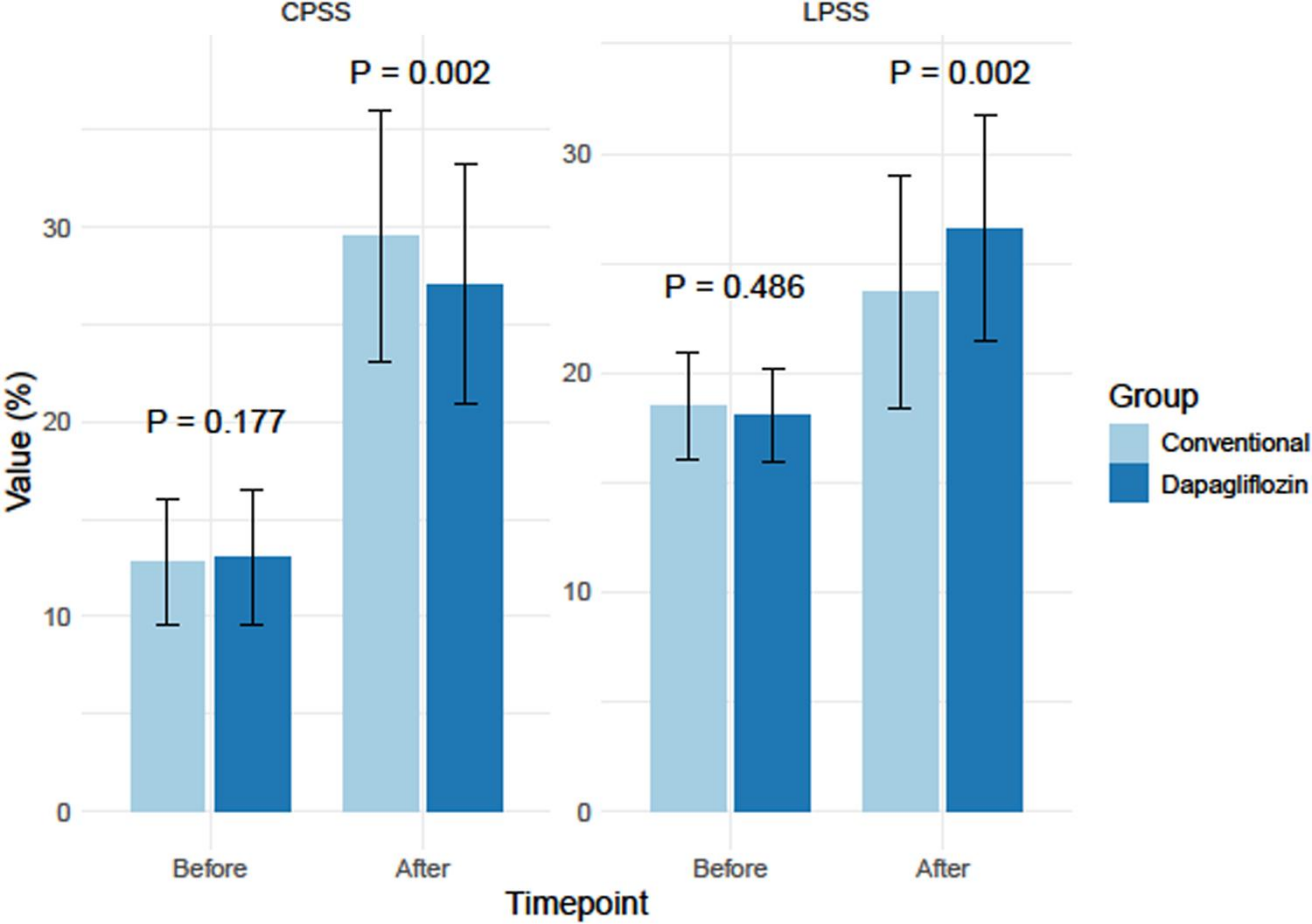
Comparison of left ventricular myocardial motion parametric between the two groups. LPSS, longitudinal pre-stretch peak strain; CPSS, circumferential pre-stretch peak strain.

As presented in Figure 1, there were no statistically significant differences in baseline left ventricular myocardial motion parameters between the conventional treatment group and the dapagliflozin group for longitudinal pre-stretch peak strain (LPSS) and circumferential pre-stretch peak strain (CPSS) (all *P* > 0.05). After treatment, LPSS was significantly higher in the dapagliflozin group compared with the conventional group (26.59 ± 5.13% vs. 23.68 ± 5.27%; t = 4.527, *P* < 0.001), while CPSS was significantly lower in the dapagliflozin group than in the conventional group (27.08 ± 6.19% vs. 29.51 ± 6.45%; t = 3.115, *P* = 0.002). These findings indicate greater improvement in LV myocardial longitudinal function with dapagliflozin therapy.

### 3.5 Left ventricular pressure-strain loop (LV-PSL) index

**Figure 2:**
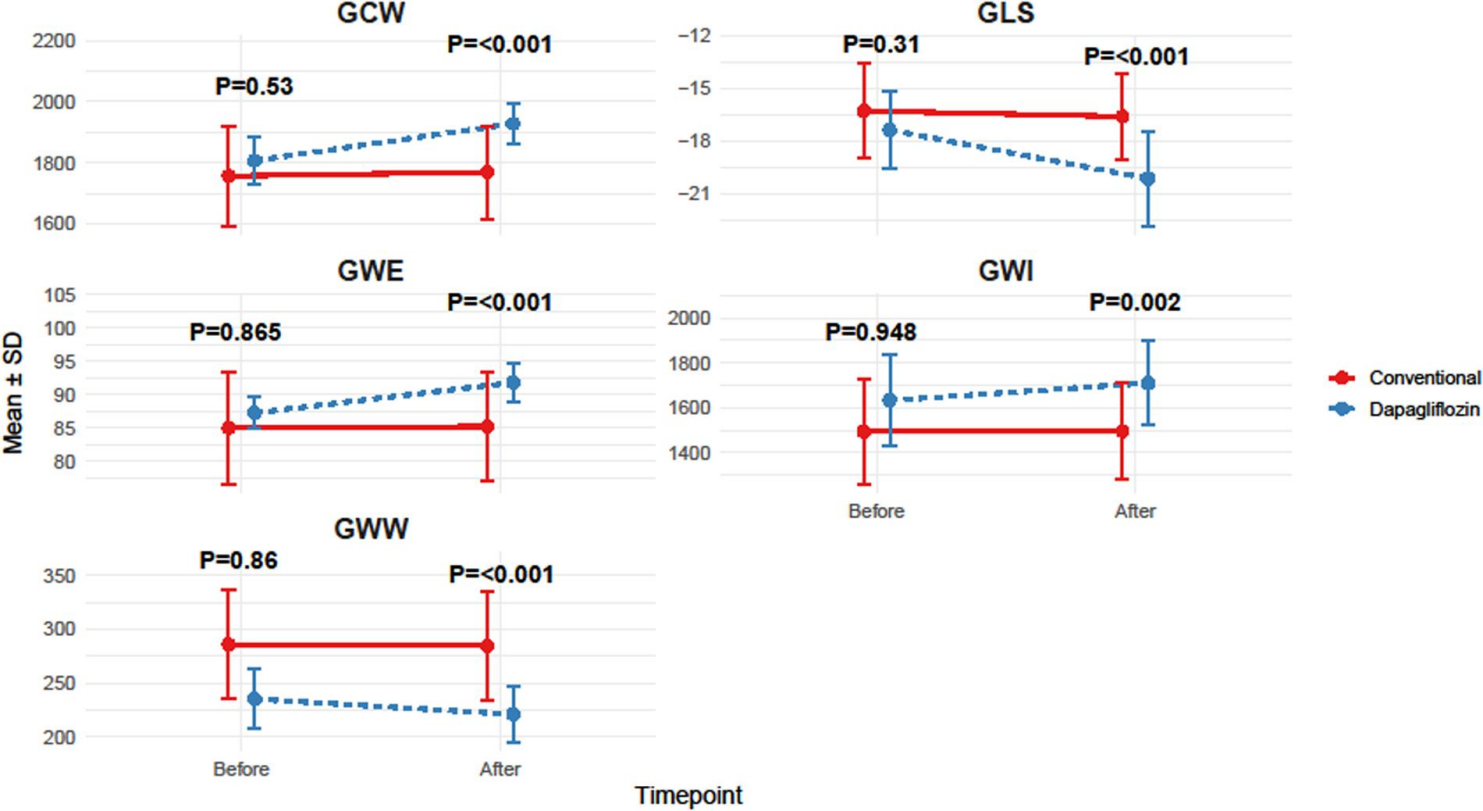
Comparison of LV-PSL indices between the two groups. GLS, left ventricular global longitudinal strain; GWW, overall wasted effort; GWE, overall work efficiency; GWI, overall work index; GCW, overall good work.

As shown in Figure 2, there were no significant differences in baseline left ventricular pressure-strain loop (LV-PSL) indices between groups, including GLS, global wasted work (GWW), global work efficiency (GWE), global work index (GWI), and global constructive work (GCW) (*P* > 0.05). After treatment, the dapagliflozin group demonstrated significantly greater improvement in GLS (−20.13 ± 2.71% vs. −17.36 ± 2.18%; t = 9.052, *P* < 0.001), higher GWE (91.82 ± 2.85% vs. 87.26 ± 2.44%; t = 13.953, *P* < 0.001), higher GWI (1709.68 ± 189.24 mmHg% vs. 1632.53 ± 203.98 mmHg%; t = 3.165, *P* = 0.002), and higher GCW (1927.57 ± 67.34 mmHg% vs. 1806.25 ± 78.21 mmHg%; t = 13.395, *P* < 0.001) compared with the conventional group. Additionally, GWW was significantly lower in the dapagliflozin group (220.76 ± 25.87 mmHg% vs. 234.92 ± 28.04 mmHg%; t = 4.234, *P* < 0.001). These results suggest that dapagliflozin provides superior enhancements in LV myocardial strain and work indices in this patient population.

### 3.6 Cardiac electrophysiological activity parameters

**Figure 3:**
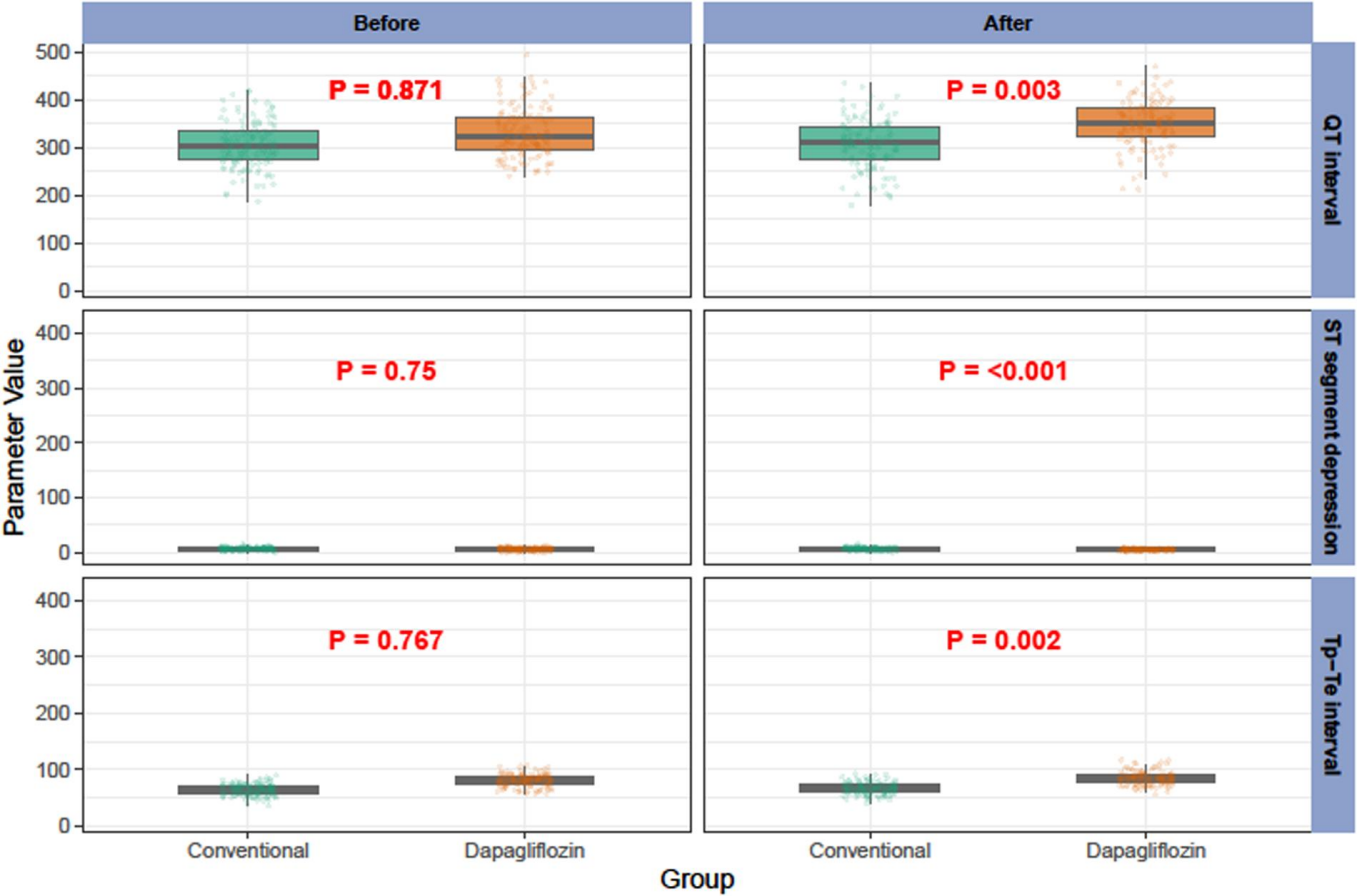
Comparison of cardiac electrophysiological activity parameters between the two groups. Tp-Te,Tpeak-Tend.

As presented in Figure 3, there were no significant differences in baseline cardiac electrophysiological activity parameters between the conventional treatment group and the dapagliflozin group, including QT interval, Tp-Te interval, and ST-segment depression (all *P* > 0.05). After treatment, the dapagliflozin group exhibited a significantly longer QT interval (347.92 ± 50.74 ms vs. 329.17 ± 50.81 ms; t = 2.985, *P* = 0.003) and longer Tp-Te interval (84.74 ± 11.29 ms vs. 80.58 ± 10.38 ms; t = 3.111, *P* = 0.002) compared with the conventional group. Additionally, ST-segment depression was significantly reduced in the dapagliflozin group after treatment (5.14 ± 2.03 mm vs. 6.43 ± 2.85 mm; t = 4.259, *P* < 0.001). These findings suggest that dapagliflozin may beneficially modulate cardiac electrophysiological activity in patients with T2DM and CHD.

### 3.7 Exercise tolerance

As shown in Table 4, there were no statistically significant differences between groups in total exercise tolerance test (ETT) time, metabolic equivalents (METs), or heart rate at MET before treatment (all *P* > 0.05). After treatment, the dapagliflozin group demonstrated a significantly longer total ETT time compared to the conventional treatment group (9.05 ± 1.69 min vs. 8.49 ± 1.44 min; t = 2.892, *P* = 0.004) and higher METs (5.09 ± 0.86 vs. 4.41 ± 0.82; t = 6.550, *P* < 0.001). Additionally, heart rate at MET after treatment was significantly lower in the dapagliflozin group (124.93 ± 5.94 bpm) than in the conventional group (131.49 ± 6.02 bpm; t = 8.870, *P* < 0.001). These data indicate that dapagliflozin treatment is associated with greater improvements in exercise tolerance and cardiopulmonary function in patients with T2DM and CHD.

**Table 4:** Comparison of indicators related to exercise tolerance between the two groups.

Indicators	Conventional Treatment group (*n* = 137)	Dapagliflozin group (*n* = 125)	t	P
Total ETT time (mins)				
Before treatment	5.37 ± 1.05	5.52 ± 1.04	1.173	0.242
After treatment	8.49 ± 1.44	9.05 ± 1.69	2.892	0.004
METs				
Before treatment	3.64 ± 0.63	3.52 ± 0.57	1.668	0.096
After treatment	4.41 ± 0.82	5.09 ± 0.86	6.550	< 0.001
Heart rate at MET (bpm)				
Before treatment	121.67 ± 5.69	122.04 ± 5.83	0.521	0.603
After treatment	131.49 ± 6.02	124.93 ± 5.94	8.870	< 0.001

ETT, exercise tolerance test; MET, metabolic equivalent of task.

### 3.8 Adverse cardiovascular events

As shown in Table 5, there were no statistically significant differences between the conventional treatment group and the dapagliflozin group in the incidence of adverse cardiovascular events, including recurrent angina pectoris, myocardial infarction, heart failure, and cardiac death (all *P* > 0.05). No deaths from cardiac causes occurred in either group during the study period.

**Table 5:** Comparison of adverse cardiovascular events between the two groups.

Events	Conventional Treatment group (*n* = 137)	Dapagliflozin group (*n* = 125)	χ^2^	P
Recurrent angina pectoris	8 (5.84%)	7 (5.60%)	0.007	0.934
Myocardial infarction	2 (1.46%)	1 (0.80%)	0.000	1.000
Heart failure	6 (4.38%)	4 (3.20%)	0.031	0.861
Death from cardiac causes	0 (0.00%)	0 (0.00%)	None	1.000

## 4 Discussion

Dapagliflozin, a sodium-glucose co-transporter 2 inhibitor (SGLT2i), has garnered considerable interest in recent years for its potential advantages beyond glycemic control in individuals with T2DM, particularly those with coexisting cardiovascular disease (17). The present study aimed to assess the effects of dapagliflozin on myocardial motion parameters and cardiac electrophysiology in patients with T2DM complicated by CHD. The findings from this investigation provide important insight into the pleiotropic cardiovascular benefits of dapagliflozin, elucidating not only clinical efficacy but also mechanistic underpinnings related to myocardial function and electrophysiological activity in this high-risk population.

The enhanced clinical efficacy of dapagliflozin observed in this study likely reflects several overlapping mechanisms by which this agent confers cardioprotection in patients with T2DM and CHD. At its core, dapagliflozin acts through inhibition of SGLT2 in the proximal renal tubule, promoting glucosuria and thereby facilitating improvements in glycemic control (17). However, the impact of dapagliflozin extends well beyond glucose lowering, encompassing a range of direct and indirect effects on cardiac structure, function, and electrophysiology (18).

One of the critical aspects of dapagliflozin's benefit lies in its ability to favorably alter myocardial metabolism (18). In T2DM and CHD, the myocardium is subjected to a milieu characterized by insulin resistance, chronic hyperglycemia, lipotoxicity, and increased oxidative stress, all of which contribute to impaired energy utilization and contractile dysfunction (19). SGLT2i therapy induces a metabolic shift toward increased ketone body utilization, providing the myocardium with a more efficient fuel source relative to glucose or free fatty acids (20). Ketones generate more ATP per molecule of oxygen consumed, thereby enhancing myocardial energetic efficiency. This metabolic remodeling supports improved contractility and may partially explain the superior recovery in echocardiographic indices—such as LVEF and GLS—and myocardial motion parameters (e.g., LPSS) in patients receiving dapagliflozin (20).

Dapagliflozin-induced natriuresis and osmotic diuresis lead to reductions in both preload and afterload, mitigating cardiac wall stress and improving hemodynamic status (21). Lower intravascular volume and decreased systemic blood pressure reduce the workload of the heart and contribute to reverse ventricular remodeling, as evidenced by the observed reductions in left ventricular end-diastolic and end-systolic diameters and decreased left atrial size (22). This hemodynamic modulation optimizes ventricular filling pressures and chamber dimensions, which, in turn, are reflected by improvements in strain-based cardiac functional metrics and diminished interventricular septal thickness (IVSd) (22). Importantly, reductions in left ventricular dimensions and improvements in myocardial strain parameters carry substantial prognostic significance, as prior studies have established that these changes are associated with favorable ventricular remodeling and a reduced risk of heart failure hospitalization, independent of changes in ejection fraction (23). In patients with diabetes and coronary artery disease, impaired GLS has been independently associated with an increased risk of hospitalization for heart failure, ventricular arrhythmias, and all-cause mortality. Likewise, GCW and GWE have emerged as sensitive predictors of adverse cardiac events, offering incremental prognostic value over traditional echocardiographic parameters (24). Notably, these structural and functional changes are achieved without significant detrimental alterations in electrolyte balance, renal function, or blood volume regulation (25).

An often underappreciated feature of SGLT2i pharmacotherapy is its role in minimizing chronic low-grade inflammation and oxidative stress—hallmarks of diabetic and ischemic cardiomyopathy (25, 26). Dapagliflozin has been shown experimentally to inhibit the activation of pro-inflammatory signaling pathways, decrease the generation of reactive oxygen species, and improve endothelial function (27). These cumulative effects help preserve microvascular integrity, reduce myocardial fibrosis, and maintain myocardial compliance (28). Such actions may underlie the observed enhancements in left ventricular myocardial motion and pressure-strain loop indices, which are sensitive indicators of subclinical myocardial dysfunction (29).

In addition to direct effects on the myocardium, dapagliflozin impacts extracardiac systems that indirectly benefit cardiac function. For example, by promoting mild weight loss, reducing visceral adiposity, and lowering blood pressure, dapagliflozin alleviates multiple cardiometabolic stressors that exacerbate CHD in diabetic individuals (30). Improvements in systemic vascular function and reductions in arterial stiffness, as described in prior literature, are likely to have contributed to better exercise tolerance and enhanced metabolic equivalents (METs) observed in the dapagliflozin cohort (31).

A particularly salient finding of this study is the beneficial modulation of cardiac electrophysiological parameters with dapagliflozin therapy. The prolonged QT and Tp-Te intervals and reduced ST-segment depression observed in the dapagliflozin group suggest a complex interplay between metabolic, structural, and electrophysiological remodeling. In patients with diabetes and coronary heart disease, chronic hyperglycemia, oxidative stress, and myocardial fibrosis often lead to heterogeneous repolarization, reflected by a shortened Tp-Te interval, which has been linked to an increased risk of ventricular arrhythmias. The prolongation of Tp-Te observed in the dapagliflozin group may therefore reflect a normalization of repolarization homogeneity, potentially mediated by reduced myocardial fibrosis, improved autonomic tone, and inhibition of the sodium-hydrogen exchanger (NHE1) (32). Indeed, experimental data indicate that SGLT2i agents may reduce arrhythmogenesis by attenuating fibrosis, modulating myocardial ion channel expression, and suppressing adverse autonomic remodeling. Recent clinical studies have further supported these electrophysiological benefits, demonstrating that SGLT2 inhibitors improve the cardio-electrophysiological balance index and reduce atrial electromechanical delay in patients with type 2 diabetes (33, 34). Moreover, the observed decrease in ST-segment depression likely reflects improved myocardial perfusion and a reduction in subendocardial ischemia, secondary to the hemodynamic and metabolic improvements described above.

The exercise tolerance benefits observed in the dapagliflozin group are likely multifactorial in origin. Enhanced ventricular performance, improved systemic oxygen delivery, and more effective substrate utilization all contribute to greater exercise capacity (35). The reduced heart rate at comparable MET levels following dapagliflozin therapy points to improved cardiac efficiency and autonomic regulation, both of which are highly relevant to morbidity and prognosis in CHD patients with T2DM (36).

Importantly, the lack of increased adverse cardiovascular events—such as recurrent angina, myocardial infarction, heart failure, or cardiac death—reinforces the cardiovascular safety of dapagliflozin in this population. The specific types of events observed were predominantly recurrent angina pectoris and heart failure, with no cases of cardiac death in either group. The numerically lower incidence of heart failure in the dapagliflozin group, while not statistically significant, aligns with the established heart failure benefits of SGLT2 inhibitors reported in large-scale trials. These reassuring safety findings are consistent with major outcome trials in T2DM (such as DECLARE-TIMI 58 and EMPA-REG OUTCOME), which report a neutral or protective effect of SGLT2i therapy on serious cardiovascular endpoints, including arrhythmic events and sudden cardiac death.

Mechanistically, the multifaceted cardioprotective effects of dapagliflozin can be attributed to the intersecting contributions of metabolic regulation, hemodynamic unloading, antifibrotic and anti-inflammatory actions, improved endothelial and vascular function, and potential modulation of neurohumoral activation (37). In CHD, where the myocardial substrate is frequently compromised by ischemia, hypertrophy, and increased wall stress, these protective effects are of particular clinical significance (38). The improvement in strain-based indices such as GLS, GWE, and GCW observed in this cohort provides mechanistic evidence in support of dapagliflozin's ability to ameliorate both subclinical myocardial dysfunction and overt contractile impairment (39).

There are also emerging hypotheses regarding direct cardiac effects of SGLT2i mediated by inhibition of the cardiac sodium-hydrogen exchanger (NHE1), reduced cytosolic sodium and calcium overload, and enhanced mitochondrial function (40). While these molecular mechanisms require further investigation in the clinical context, they offer additional biological plausibility to the cardiac-specific benefits observed in this study.

Some limitations must be acknowledged. As a retrospective, single-center study, there may be unmeasured confounders affecting the generalizability of the results. Furthermore, the relatively short duration of the intervention (4 weeks) limits assessment of long-term outcomes and adverse effects, including rare cardiovascular events or progressive cardiac remodeling. Additionally, formal effect size calculations were not performed, which may limit the interpretability of the magnitude of treatment effects beyond statistical significance; future studies should incorporate such metrics to facilitate clinical translation. Prospective randomized controlled trials with extended follow-up are warranted to confirm and expand on these findings and to further elucidate the implications of dapagliflozin in the broader spectrum of diabetic patients with various stages of cardiovascular disease.

## 5 Conclusion

In conclusion, the present study contributes to a growing body of evidence supporting the multifactorial cardiac benefits of dapagliflozin in patients with T2DM and CHD. By improving myocardial motion parameters, enhancing pressure-strain loop indices, optimizing electrophysiological activity, and supporting greater exercise tolerance without a rise in adverse cardiovascular events, dapagliflozin delineates a promising therapeutic strategy for this vulnerable population. The underlying mechanisms appear to reflect a synergy of metabolic, hemodynamic, anti-inflammatory, and potentially direct myocardial actions, altogether underscoring the paradigm shift toward cardiovascular risk modulation in diabetes management.

## Data Availability

The original contributions presented in the study are included in the article/Supplementary Material, further inquiries can be directed to the corresponding author.
